# Prolonged exposure for the treatment of Spanish-speaking Puerto Ricans with posttraumatic stress disorder: a feasibility study

**DOI:** 10.1186/1756-0500-4-415

**Published:** 2011-10-17

**Authors:** Mildred Vera, María L Reyes-Rabanillo, Deborah Juarbe, Coralee Pérez-Pedrogo, Alicia Olmo, Rafael Kichic, William F Chaplin

**Affiliations:** 1 Center for Evaluation and Sociomedical Research and Department of Health Services Administration, School of Public Health, Medical Science Campus, University of Puerto Rico, San Juan, Puerto Rico; 2 Veterans Affairs Caribbean Healthcare System, San Juan, Puerto Rico; 3 Center for Evaluation and Sociomedical Research, School of Public Health, Medical Science Campus, University of Puerto Rico, San Juan, Puerto Rico; 4 Anxiety Clinic, Institute of Cognitive Neurology, Buenos Aires, Argentina; 5 Department of Psychology, St. John's University, New York, USA

## Abstract

**Background:**

Most of the empirical studies that support the efficacy of prolonged exposure (PE) for treating posttraumatic stress disorder (PTSD) have been conducted on white mainstream English-speaking populations. Although high PTSD rates have been reported for Puerto Ricans, the appropriateness of PE for this population remains unclear. The purpose of this study was to examine the feasibility of providing PE to Spanish speaking Puerto Ricans with PTSD. Particular attention was also focused on identifying challenges faced by clinicians with limited experience in PE. This information is relevant to help inform practice implications for training Spanish-speaking clinicians in PE.

**Results:**

Fourteen patients with PTSD were randomly assigned to receive PE (n = 7) or usual care (UC) (n = 7). PE therapy consisted of 15 weekly sessions focused on gradually confronting and emotionally processing distressing trauma-related memories and reminders. Five patients completed PE treatment; all patients attended the 15 sessions available to them. In UC, patients received mental health services available within the health care setting where they were recruited. They also had the option of self-referring to a mental health provider outside the study setting. The Clinician-Administered PTSD Scale (CAPS) was administered at baseline, mid-treatment, and post-treatment to assess PTSD symptom severity. Treatment completers in the PE group demonstrated significantly greater reductions in PTSD symptoms than the UC group. Forty percent of the PE patients showed clinically meaningful reductions in PTSD symptoms from pre- to post-treatment.

**Conclusions:**

PE appears to be viable for treating Puerto Rican Spanish-speaking patients with PTSD. This therapy had good patient acceptability and led to improvements in PTSD symptoms. Attention to the clinicians' training process contributed strongly to helping them overcome the challenges posed by the intervention and increased their acceptance of PE.

## Background

### Exposure therapy and posttraumatic stress disorder

Prolonged exposure (PE), a form of cognitive behavioral therapy, has received strong support for of the treatment of posttraumatic stress disorder (PTSD) [1-3]. A committee convened by the Institute of Medicine (IOM) to assess evidence about the efficacy of treatments for PTSD concluded that while the evidence was inadequate for most psychological and pharmacological treatment modalities, the efficacy of exposure therapies for treating PTSD was strongly supported [4]. The superior efficacy of PE for treating PTSD has been demonstrated across a wide range of traumatized populations, including victims of rape, violent assault, child abuse, combat-related trauma, motor vehicle accidents, and disasters [1,5-7].

Although strong empirical evidence sustains the efficacy of PE for PTSD, the adoption of exposure therapy in clinical practice has been slow [8-11]. Lack of adequate training has been identified as a contributing factor to the low use of exposure therapy by clinicians in treating PTSD [12]. Other common obstacles have been discussed in the literature, including beliefs that exposure therapy is aversive, it retraumatizes the victim, it does not generalize to clinical settings, and it makes the therapist look more like a technician than a supportive clinician by relying on a treatment manual [13-16].

Studies that evaluate the outcomes of exposure therapy have generally disconfirmed clinicians' concerns about exposure therapy [13,14,17-19]. However, according to Gunter and Whittal [20], it should not be assumed that knowledge of empirical data sustaining the efficacy of exposure is enough to ease clinicians' concerns. These authors noted that the use of exposure requires that clinicians have trust in the intervention, feel comfortable administering it, and have confidence in their ability to address client reactions during exposure treatment. There is common agreement that brief courses and training sessions are not sufficient to change clinician practice behaviors. To prepare clinicians emotionally and technically for the use of exposure therapy, a combination of didactics, ongoing supervision that specifically addresses clinician concerns during the administration of treatment, and peer consultation is recommended [18,20,21]. In the training process, Hembree and Cahill [18] identified four cornerstones that are fundamental to the practice of good exposure therapy: conceptualization, alliance, rationale, and effective implementation. They indicate that it is essential that clinicians develop a clear understanding of the conceptual model upholding exposure therapy. The second cornerstone, alliance, emphasizes the importance that therapists provide support, encouragement, and warmth while implementing a manualized treatment. This is extremely important in helping clients overcome their fears. The third cornerstone entails providing clients with a clear rationale for treatment. It is critical that the rationale is compelling and makes sense to the client. The fourth cornerstone involves the effective implementation of exposure techniques. Adjusting exposure interventions to each client's fear structure is central to this treatment. Hembree and Cahill [18] also highlighted the importance of addressing therapists' concerns about exposure therapy throughout training, as well as possible reluctance to use treatment manuals. They describe the effective use of manualized treatments as an "art" built on knowledge and practice.

Most of the empirical studies that support the efficacy of prolonged exposure (PE) for the treatment of posttraumatic stress disorder (PTSD) have been conducted on white mainstream English-speaking populations. Although high PTSD rates have been reported for Puerto Ricans [22-24], the appropriateness of PE for this population remains unclear. The IOM committee on treatment of PTSD [4] noted that even though it could be expected that psychotherapies present special challenges in different cultural groups, they were unable to comment because "the evidence is mostly silent on the acceptability, efficacy, or generalizability of treatment in ethnic and cultural minorities, as few studies stratified results by ethnic background". As such, the purpose of this article is to build on the dearth of treatment studies on ethnic minority groups by examining the feasibility of providing PE to Spanish speaking Puerto Ricans with PTSD. Particular attention is also focused on identifying challenges faced by clinicians with limited experience in PE. This information is relevant to help inform practice implications for training Spanish-speaking clinicians in PE.

## Methods

### Participants

Patients were eligible for the study if they were aged 18 - 65 years, met the diagnostic criteria for PTSD and scored at least 45 on the Clinician-Administered PTSD Scale, could speak Spanish fluently, and were competent to provide informed consent. We excluded patients if they were in potentially life-threatening circumstances (i.e. serious suicide risk or serious illness), had a history of bipolar, schizophrenia or psychotic disorder, had abused drugs or alcohol during the past three months, had moderate to severe traumatic brain injury or were applying for or receiving disability benefits. Patients receiving psychotherapy for PTSD or those who had initiated or changed psychotropic medication within two months of randomization were not eligible. However, patients could participate in the study if psychotropic medications had been kept constant for at least two months. If they were randomized to PE, doses of psychotropic medications were not changed during the study.

Patients were recruited by referral from mental health providers in a general health setting located in Puerto Rico. After exclusion criteria were examined in medical records, potentially eligible patients were invited to participate in a face-to-face interview. Patients who complied with eligibility conditions were randomly assigned to PE (n = 7) or usual care (UC, n = 7). All participants provided informed consent and all study procedures received institutional review board approval.

### Therapist training and supervision

Five therapists with doctoral degrees administered PE therapy. While all therapists had experience with cognitive behavior therapy, they had no previous experience with PE. The five study patients served as the therapists' training cases. During the initial phase of the study, therapists participated in two training workshops. All therapists received ongoing supervision throughout the study. Therapy sessions were video recorded and viewed by our Spanish-speaking supervisor, who provided written and oral feedback. Supervision was provided in 2 - 2 1/2 hour weekly meetings held by videoconference or teleconference. Activities included didactic instruction, feedback on therapy sessions, active discussion of the therapist's concerns during the implementation of PE, role-play practice of specific techniques, and direction in applying the concepts and principles of PE. Therapy cases were initiated sequentially, facilitating role-modeling and peer consultation among therapists.

### Study measures

Assessments were administered at baseline, week 8 (mid-treatment), and week 16 (post-treatment) by interviewers blind to intervention status. Of the 14 patients enrolled, a total of 5 PE patients (71%) and 7 UC patients (100%) completed all three assessments. The other two PE patients completed only the baseline assessment. Assessments included the Clinician-Administered PTSD Scale and sociodemographic information including age, sex, marital and employment status, and education.

#### Clinician-Administered PTSD Scale

The Clinician-Administered PTSD Scale (CAPS) [25] is a semi-structured interview designed to assess PTSD diagnostic status and symptom severity. The CAPS includes a total of 30 items. Seventeen items focus on the 17 PTSD symptoms, assessing five symptoms of reexperiencing (Criterion B), seven of avoidance or numbing (Criterion C), and five of hyperarousal (Criterion D). For each item, standardized questions and probes are provided. Each item is scored on a separate five-point scale (0 - 4) to determine the frequency and intensity of individual PTSD symptoms. We determined PTSD diagnosis with the CAPS following the "1/2" rule, which stipulates that a symptom is present if it occurs at least monthly with moderate intensity [26]. A total score is obtained by summing the frequency and intensity scores for each of the 17 symptom items. Accordingly, total scores can range from 0 to 136. A cutoff point of 45 provides a good balance between sensitivity and specificity and is used as a threshold criterion for PTSD [27-30]. The CAPS has been translated into several languages including Spanish. Psychometric studies of the English [26] and Spanish [31] versions of the CAPS sustain their validity and reliability.

### Treatment conditions

#### Prolonged exposure

PE therapy was delivered in 15 weekly sessions each lasting 90 - 120 minutes, according to a culturally adapted Spanish version of the PE manual (Foa EB, Hembree EA, Dancu CV: Prolonged exposure manual revised version, unpublished). While maintaining fidelity to the English PE manual, the Spanish version includes language, idioms, and examples relevant to Latino culture, placing special emphasis on the attainment of conceptual and cultural equivalence. Latino core values and norms are highly oriented towards family. Although most cultures strongly value family relationships, Latinos appear to have a more interdependent sense of themselves, which may contribute to assuming a more active role in the care and support of family members when they are ill [32]. To take into consideration this element of Latino culture within PE, we designed an introductory session targeted at participants and their spouses or partners. The main purpose of this session was to provide the patient's spouse or partner with an overview of PTSD and the main goals and methods of exposure therapy. Exposure homework assigned throughout therapy can place significant demands on patients. Support provided by significant others can contribute to facilitate the patient's progress. This session was optional; patients decided whether they wanted to invite their spouses or partners after the goals of the session had been discussed.

The first PE session focused on information gathering and the discussion of treatment rationale and program overview. The second session included information about common reactions to trauma and an introduction to repeated in vivo exposure. A hierarchy for in vivo exposure was established, from least to most distressing stimuli. The assignment of exposure homework began in this session. Session 3 focused on discussion of the rationale and practice of imaginal exposure. Patients were asked to close their eyes, revisit the trauma memory and recount the memory aloud in the first-person and present tense. Imaginal exposure was repeated several times per session, for a period of approximately 45 minutes. In this session the patient's experience with in vivo exposure homework was explored. They were also assigned additional homework that involved listening daily to the audiotape of the imaginal exposure. If this session content could not be completed within the assigned time period, the discussion continued during the following session. Sessions 4 - 14 included revision of homework assigned in the previous session, imaginal exposure lasting 30 - 45 minutes, discussion of imaginal exposure to facilitate emotional processing of the problematic traumatic memories, and assignment of in vivo and imaginal exposure homework. In addition to these activities, the final session included a discussion of the patient's progress and future plans.

#### Usual care

Patients assigned to the UC group were referred to a mental health provider within the health care setting. They also had the option of self-referring to a mental health provider outside the study setting. In most cases, usual care for PTSD provided at the study setting involved pharmacological medication or psychotherapy.

### Statistical analysis

We examined whether the demographic and clinical characteristics of patients in PE and UC were equivalent at baseline by using *t *tests for continuous data and Fisher's exact test for categorical data. Given the nature of this study we first examined the data on a case-by-case basis. Next, data were pooled and analyzed. We used mixed-effects regression to evaluate longitudinal outcomes. Because there was a substantial mean difference between the treatment and control groups on the outcome measure at baseline, we controlled for this difference by including the baseline scores as a covariate in the analysis. One patient had a score of 0 on the CAPS at the post-treatment assessment. Zero is a possible value on this scale and is valid. Although this score does not meet criteria as an outlier, we winsorized [33] the distribution to reduce the extremity of this score so that the results were not unduly influenced by this one participant. Plots of the outcome measure over time showed curvilinear patterns, so we evaluated both a linear and a quadratic trend. We represented the trends in the data using orthogonal contrast codes for the linear (-0.707, 0, and 0.707) and quadratic (0.408, -0.816, 0.408) trends applied to baseline, week 8 and week 16, respectively. We dummy-coded the treatment groups as 0 = control and 1 = experimental. Differences in the treatment groups in linear and quadratic change over time were assessed by testing the treatment by time linear and treatment by time quadratic trends, respectively. Although our preference with clinical trial data is to estimate a robust unstructured covariance matrix for the error term, the small sample size and the necessity of including the baseline scores as a covariate prevented this approach from converging. We therefore estimated a simpler autoregressive covariance structure with homogenous variances in these analyses. We used a p value of *<*0.05 to detect statistically significant differences. The analyses were conducted using SPSS software, version 16.0.

## Results

### Patient characteristics

Of seven patients randomized to PE, one dropped out after randomization and did not attend any session, one discontinued treatment after five sessions owing to serious physical health complications unrelated to PE delivery, and five completed treatment. Given the nature of this study, the five PE patients who completed treatment were included in data analyses.

The PE and UC groups were equivalent (p > 0.05) on all baseline variables with the exception of pre-treatment mean CAPS scores. CAPS scores were higher for UC (*M *= 73.29) than PE (*M *= 53.20) patients. The mean age of the participants was 45.8 years, 75% were married or cohabiting, and all were men. Seventy-five percent of the patients were employed and two-thirds had taken some college studies.

### Treatment received by PE and UC patients

Attendance at PE sessions was excellent among treatment completers, with an average of 15 sessions, showing that each patient attended all 15 sessions offered. Four out of the five PE patients who completed treatment invited their spouses or partners to the optional introductory session focused on providing an overview of PTSD and the main goals and methods of PE treatment. All patients assigned to UC received mental health treatment. They were seen by a mental health specialist at least once (range, 1 - 9), during the 16 week period beginning with the date of randomization. Four patients received pharmacological treatment, one patient received psychotherapy, and two patients received both pharmacological and psychological treatment.

### Outcomes

Table 1 displays individual mean CAPS severity scores at each assessment period and the percentage change from pre-treatment to the other assessment periods. A visual assessment of the data revealed that PTSD symptoms increased from pre- to mid-treatment for four out of the five PE patients. However, by post-treatment, reductions of 25% or more of the pre-treatment PTSD symptoms were identified for three of these patients. The post-treatment CAPS severity score for the other patient returned to pretreatment level. In contrast, the CAPS severity scores increased from pre- to post-treatment for almost half of the patients in UC.

**Table 1:** Individual mean CAPS scores by assessment and percentage change from pre-treatment to other assessments

Patient	Pre-treatment	Mid-treatment	Post-treatment	Change pre- to mid-treatment	Change pre- to post-treatment
PE					
1	57	86	43	+51%	-25%
2	67	67	62	0%	-7%
3	48	70	48	+46%	0%
4	46	61	28	+33%	-39%
5	48	54	0	+13%	-100%
					
Mean	53.20	67.60	36.20		
SD	8.81	11.97	23.61		
					
UC					
1	85	70	81	-18%	-5%
2	97	99	109	+2%	+12%
3	64	31	49	-52%	-23%
4	76	85	94	+12%	+24%
5	50	69	70	+38%	+40%
6	89	88	81	-1%	-9%
7	52	71	49	+37%	-6%
					
Mean	73.29	73.14	76.14		
SD	18.42	21.55	22.2		

A 15-point change or more in CAPS scores can be used to indicate clinically significant changes in PTSD symptoms [26]. At post-treatment, two out of five (40%) PE patients and one out of seven UC patients (14.3%) showed a clinically significant reduction in PTSD symptoms. Two PE patients no longer complied with DSM-IV PTSD diagnostic criteria at the post-treatment assessment, while no change in diagnostic status was observed among UC patients.

**Figure 1:**
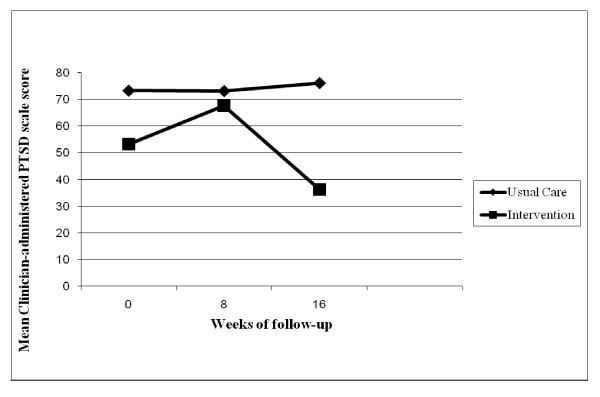
**Mean Clinician-Administered PTSD Scale Scores by Treatment Assignment**.

Outcome was further assessed by mixed-effects regression models using CAPS data from pre-treatment, mid-treatment, and post-treatment assessments (Table 2). Models were fit with both linear and quadratic time effects. Pre-treatment CAPS score was included as a covariate. The critical time by group interaction was significant for a quadratic function (p = 0.01) and the linear time by treatment interaction approached conventional statistical significance (p = 0.07). Given the small sample size it is not surprising that the effect size estimates were substantial with an estimated linear decrease of -14.0 points and a quadratic "spike" of -20 points in the treatment group compared to the control group. The plot of the estimated means from these analyses is shown in Figure 1. The interaction effect was, specifically, that the PE group evidenced a larger reduction in PTSD symptoms between pre-treatment and post-treatment relative to the UC group.

**Table 2:** Mixed-effects regression estimates for patients assigned to Prolonged Exposure or Usual Care

	Outcomes
	
Effect	PTSD Severity
Time	
*β*	2.02
*F*	0.42
*df*	28.51
*p value*	0.68
	
Time^2^	
*β*	1.28
*F*	0.33
*df*	15.30
*p value*	0.75
	
Treatment	
*β*	-3.07
*F*	-0.46
*df*	11.66
*p value*	0.66
	
Time × Treatment Interaction	
*β*	-14.04
*F*	-1.90
*df*	28.51
*p value*	0.07
	
Time^2 ^× Treatment Interaction	
*β*	-19.99
*F*	-3.29
*df*	15.30
*p value*	0.01

## Discussion

Although Puerto Ricans have been found to be at increased risk for PTSD, there is little information about the applicability of PE therapy in this group. The results of this initial study with Puerto Rican patients support the feasibility and acceptability of PE therapy. Almost all of the patients who initiated PE therapy completed treatment. Even though an increase in PTSD symptoms was observed in the mid-treatment assessment, PE therapy had reduced PTSD symptoms to a greater degree than usual care by post-treatment. In addition to sustaining the feasibility of implementing the intervention, the significant reduction in PTSD symptoms after PE therapy suggests that PE is promising for the treatment of Puerto Rican patients with PTSD.

Contrary to the widespread perception that symptom exacerbation can increase treatment dropout, this was not evidenced in our study. Findings revealed a high retention rate for patients who initiated PE; all patients except one who experienced a serious physical health condition attended all 15 sessions offered. The inclusion of an introductory session to facilitate that patient's partners understand the main goals and methods of PE treatment might have contributed to these excellent retention rates. Data from prior focus groups with Latino patients revealed that support provided by significant others was instrumental in the patients process of seeking and continuing mental health care. The introductory session was developed to allow discussing with patients' partners the affective intensity required from patients in the processing of a trauma, the significant demands for time placed by homework assignments, and the potential for treatment dropout. Patients reports about the strong support provided by their partners throughout treatment suggest that the information discussed in the introductory session had the desired effect and could help account for the high treatment retention rate. The issue of Latino family support for retention in PE treatment is a relevant one for future research.

Several limitations should be noted in interpreting these results. First, the small sample size does not allow the findings to be generalized to other Puerto Ricans with PTSD. Second, the lack of follow-up assessments limits information about treatment effects over time. It is not possible to establish whether treatment gains were maintained or whether significant improvements could have been seen over a follow-up period. Third, all patients who participated in the study were men; it is necessary that future studies include women. Fourth, the PE patients included in the study were the first training case for each clinician. However, it is notable that even though the clinicians had limited PE experience, the study results are very encouraging.

In the process of providing therapy, the clinicians were faced with a range of challenges. Careful attention to their training process contributed strongly to helping them overcome the challenges posed by the intervention and increased their acceptance of PE. Following we present some examples of the methodological or practical challenges encountered and the lessons learned, which could help inform practice implications for training clinicians in PE.

An important factor to take into consideration in preparing clinicians to deliver an intervention competently is the quality of the content and design of the training program [34]. As a first step in this direction, the study clinicians participated in a four-day workshop aimed at facilitating and developing their understanding of the conceptual background and principles that sustain PE, rather than focusing only on techniques. In addition to reading materials and didactic presentations, the workshop included active training methods such as videos, role modeling, practice and role-play opportunities, and interaction among learners. These training strategies have been associated with improved knowledge and attitudes, expanded skill sets, and demystification of common trauma beliefs [8,18,21,34,35].

Ongoing supervision is considered critical for improving clinicians' competence and skills and increasing confidence in their ability to implement exposure therapy [20]. The clinicians in our study agreed that further assistance was needed to develop the competence and confidence to put the skills learned in the workshop into practice. After the initial training, it is important that therapists receive ongoing supervision while they administer the treatment. One of the challenges we experienced in the process of implementing our study was identifying a PE clinical supervisor with both the necessary credentials and command of the Spanish language. We could not identify a qualified supervisor in Puerto Rico; however, we were able to establish collaboration with a Spanish-speaking PE supervisor in Latin America and a consultant in the US. Santisteban and collaborators [35] noted that supervisors who are viewed by therapists as supportive and are able to foster a sense of teamwork are more likely to establish a successful supervisory process. In setting up the supervision process in our study, significant efforts were directed at addressing these factors. After our Spanish-speaking supervisor was identified, a face-to-face training workshop was planned at the initial stage of the project. This activity established the basis for a receptive, supportive environment in which open discussion and collaboration were emphasized. Cultural and organizational issues relevant to implementing the intervention were also discussed. The group cohesion and personal relationships resulting from this activity contributed to the progress of subsequent supervisory meetings.

Clinicians' fears that exposure to trauma memories can have harmful effects on patients or cause them to drop out from treatment have contributed to limiting the use of PE [18]. The clinicians in our study were aware that these concerns have not been supported by research evidence. However, the report of symptom increase among PE patients in our study proved to be worrying for them. Owing to their limited experience in PE therapy, patient reports about exposure-induced symptom exacerbation raised the therapists' concerns about doing harm to patients. The support and direction provided by study supervisors, as well as in depth discussions about the conceptual framework and principles sustaining exposure exercises, were instrumental in facilitating the development of the therapists' skills needed to address patients' reports adequately. Furthermore, since supervision was provided in group format, this provided the clinicians with the opportunity to share their concerns with peers, realize and understand that they were not unique in their initial hesitancy, and learn from each other's experiences. If it were not for the guidance provided by the clinical supervisors, we could have jumped to the conclusion that we were being insensitive to patients' needs or that PE was not tolerable for our patient population. As evidenced by our study findings, patients remained in treatment and exposure had a positive impact in reducing PTSD symptoms.

Another factor that contributed to the encouraging results observed in this study was the clinicians' receptiveness and positive attitude towards the use of manualized treatments. Concerns have been raised that the use of treatment manuals limits therapists' clinical creativity and negatively impacts the therapeutic relationship. In addressing these concerns, Hembree & Cahill [18] acknowledge that learning to use a treatment manual can be very demanding. They specify that the process requires that the therapist: (1) have a clear understanding of the conceptual model, (2) invest substantial time in getting to understand the treatment manual thoroughly, and (3) apply the treatment while adjusting the intervention to the client's responses. In addition, they highlight that throughout treatment the clinician must attend to the basic therapy and interpersonal skills that affect the patient-therapist relationship and treatment process. The experience of our study clinicians was that training activities and implementation of the PE protocol required a high level of commitment. Learning exposure therapy was demanding and time-consuming; each therapist attended weekly supervision meetings consistently and spent time preparing for sessions and reviewing videos. It was our experience that the clinicians' motivation and commitment to learning a new model was fundamental to the effective implementation of PE.

## Conclusions

PE appears to be viable for treating Puerto Rican Spanish-speaking patients with PTSD. It showed good patient acceptability and led to improvements in PTSD symptoms. Additional research examining the efficacy of PE with this clinical population is warranted. The present study also highlighted significant practical and methodological challenges faced by clinicians with limited experience in PE. Attention to the training process contributed strongly to helping clinicians overcome the challenges posed by the intervention and increased their acceptance of PE. These findings are relevant to inform practice implications for training Spanish-speaking clinicians in PE.

## Competing interests

The authors declare that they have no competing interests.

## Authors' contributions

MV was responsible for the conception, design, and implementation of the study and wrote the first and final drafts of the manuscript. MLRR contributed to the conception and design of the study and its implementation. DJ, CPP, AO and RK assisted with the study design and its implementation. WFC assisted with the analysis and interpretation of data. All authors revised the manuscript critically and approved the final draft.

## Funding/support

This project was supported by grant SC1MH090599 from the National Institute of General Medical Sciences and grant 1U54RR026139-01A1 from the National Center for Research Resources (NCRR).
